# Clinical and molecular characteristics of *Klebsiella pneumoniae* ventilator-associated pneumonia in mainland China

**DOI:** 10.1186/s12879-016-1942-z

**Published:** 2016-10-26

**Authors:** Si Guo, JingJing Xu, YanShuan Wei, JunHong Xu, Yi Li, Rui Xue

**Affiliations:** 1 Department of Clinical Microbiology, Henan Provincial People’s Hospital, Zhengzhou, China; 2 Department of Infection control, Zhengzhou University People’s Hospital, Zhengzhou, China; 3 Department of Pathology, The First Affiliated Hospital of Zhengzhou University, Zhengzhou, China; 4 Department of Clinical Laboratory, Henan No. 2 Provincial people’s Hospital, Zhengzhou, China; 5 Department of Key Laboratory, The First Affiliated Hospital of Zhengzhou University, Zhengzhou, China

**Keywords:** *Klebsiella pneumoniae*, Ventilator-associated pneumonia, Hypermucoviscosity, Virulence determinant

## Abstract

**Background:**

*Klebsiella pneumoniae* is a prominent nosocomial pathogen that accounts for up to 10 % of all hospital-acquired infections. It is a frequent cause of ventilator-associated pneumonia (VAP). The purpose of this study was to investigate the clinical characteristics of *K. pneumoniae-*associated VAP and the molecular characteristics of *K. pneumoniae* strains.

**Methods:**

We retrospectively reviewed 70 mechanically ventilated patients with *K. pneumoniae* isolated. All *K. pneumoniae* strains were examined to determine hypermucoviscosity (HV) phenotype, capsular serotypes, virulence genes, multilocus sequence typing and antimicrobial susceptibility.

**Results:**

Hypermucoviscosity was found in 14 of 70 (20 %) isolates of *K. pneumoniae*. Among the 70 patients, 43 cases (61.4 %) developed VAP. Furthermore, VAP was more frequently induced by HV-positive *K. pneumoniae* (14/14, 100 %) than by HV-negative strains (29/56, 51.7 %). HV-positive *K. pneumoniae-*associated VAP patients were more inclined to develop bacteremia and had a higher mortality rate than HV-negative strains VAP patients. Antibiotic resistance was more frequent in HV- negative strains- than in HV- positive strains-infected patients. The prevalence of *rmpA* and aerobactin genes were 85.7 % and 85.7 % respectively, and serotypes K1 and K2 accounted for 14.3 % and 28.6 % of the hypermucoviscosity strains, respectively. Strains carrying *rmpA* and aerobactin genes were significantly associated with HV-phenotype, and *rmpA* and aerobactin coexisted in HV-positive strains. Multilocus sequence typing analysis identified 24 different sequence types from *K. pneumoniae* VAP samples.

**Conclusions:**

HV-phenotype is the major virulence determinant for mechanically ventilated patients. There was a specific sequence typing (ST) distribution between HV-positive and HV-negative strains.

## Background

Ventilator-associated pneumonia (VAP) is defined as nosocomial pneumonia occurring in a patient after 48 h of mechanical ventilation. The occurrence rate of VAP is reportedly 9–27 %, and mortality reaches 20–50 % [1–3]. Common causative pathogens of VAP include gram-negative bacteria such as *Pseudomonas aeruginosa*, *Klebsiella pneumoniae*, and *Escherichia coli* and gram-positive bacteria such as *Staphylococcus aureus* [4–9]. *K. pneumoniae* is a common pathogen responsible for both community-acquired and nosocomial infections [10]. It also causes miscellaneous infections such as meningitis, septicemia, purulent abscesses, and pneumonia. Previous investigations have implicated *K. pneumoniae* in 7–12 % of nosocomial pneumonia in intensive care units in the United States [11, 12].

It has been reported that the HV-positive phenotype, certain serotypes, and the presence of *rmpA* and aerobactin genes are virulence determinants in *K. pneumoniae* infection [13–17]. However, there have been few reports on the specific roles of these factors in VAP in mainland China. In the present study, we used isolates collected from mechanically ventilated patients to delineate the clinical characteristics of *K. pneumoniae*-associated VAP and molecular characteristics of *K. pneumoniae* strains observed over a 2-year period.

## Methods

### Hospital setting and study population

The Henan Provincial People’s Hospital is a 3900 bed tertiary care hospital with 6 ICU wards with an approximate annual admission of 1600 ICU inpatients. *K. pneumoniae* strains were collected via endotracheal aspiration from mechanically ventilated patients with suspected pneumonia and stored at −80 °C before use. Medical records of patients from whom the collected strains were isolated were reviewed between January 2012 and August 2014. VAP was diagnosed in these patients who fulfilled both the clinical and microbiological criteria. The clinical criteria for the diagnosis of VAP are the presence of a new pulmonary infiltration on chest radiography plus at least two of the following: fever above 38 °C, purulent secretions, and leukocytosis or leucopenia [18]. The microbiological criteria are quantitative tracheal aspirate culture with ≥10^5^ CFU/mL and positive gram stain (>10 polymorphonuclear cells/low-power field and ≥1 bacteria/oil immersion field with or without intracellular bacteria) [19–21]. The VAP diagnosis was reconfirmed by two infectious diseases specialists independently. Polymicrobial infections were excluded from the analysis.

### Data collection and microbiologic analysis

The following clinical information was collected for each patient: demographic characteristics, VAP diagnosis, reasons for mechanical ventilation, laboratory data, and chest radiograph reports. Bacteremic VAP was diagnosed when blood and respiratory samples yielded the same microorganism and other sources of infection that could account for the bacteremia were absent. Furthermore, blood and respiratory cultures were performed within 48 h.

Outcome was defined as in-hospital mortality measured 30 days after the onset of VAP. Trauma was defined as the presence of injury in more than one body area or system or the presence of major cranial trauma alone. Chronic lung diseases included bronchiectasis, chronic obstructive pulmonary disease. The initial laboratory value was defined as that measured within 48 h of the onset of VAP.

The BD Phoenix system (Becton Dickinson, USA) was used to confirm bacterial identification. Antibiotic susceptibility was tested with the disk diffusion method, and interpretations were made according to the guidelines of the Clinical and Laboratory Standards Institute [22]. All of the *K. pneumoniae* isolates were screened and confirmed by a double-disk synergy test for produced extended-spectrum β-lactamase (ESBL).

### Detection of HV-phenotype

For HV-phenotype determination, a standard bacteriologic loop was used to stretch a mucoviscous string vertically from a colony. The formation of a viscous string of >5 mm confirmed the HV-positive phenotype.

### Serotyping, *rmpA* and aerobactin gene detection with PCR

PCR was performed to amplify genes specific for serotypes K1/K2 and the *rmpA* and aerobactin genes as described previously [17, 23]. *K. pneumoniae* ATCC9997 (K2) was used as a control strain. A bacterial colony from an overnight culture was added to 500 μL water and boiled for 15 min to release the DNA template. PCR was performed with the following conditions: 95 °C initial denaturation for 5 min followed by 30 cycles at 95 °C for 30 s, 55 °C for 30 s, 72 °C for 90 s, and a final extension at 72 °C for 5 min.

### Multilocus sequence typing (MLST)

MLST was performed to determine the diversity of clinical *K. pneumoniae* in patients with VAP. Seven housekeeping genes (*rpoB*, *gapA*, *mdh*, *pgi*, *phoE*, *infB*, and *tonB*) were amplified via PCR with conditions and primers designated by the Pasteur Institute *Klebsiella pneumonia* MLST Database (http://bigsdb.pasteur.fr/klebsiella/klebsiella.html).

### Statistical analysis

SPSS 17.0 was used for statistical analysis. The chi-square or Fisher’s exact test was used to analyze contingency data, and continuous data were analyzed with the Student’s *t* test. A *P* value of <0.05 was considered significant, and all probabilities were two-tailed.

## Results

### Clinical characteristics of *K. pneumoniae*-associated VAP patients

The retrospective study was conducted in 70 mechanically ventilated patients with *K. pneumoniae* isolated. Among these patients, 43 cases (61.4 %) developed VAP during their ICU stay. The patients had mean ± standard deviation age of 57.0 ± 15.0 years. Thirty-two (74.4 %) were male and 11(25.6 %) were female. Fourteen (32.5 %) HV-positive strains and 29 (67.4 %) HV-negative strains were isolated from the 43 VAP patients. None of the VAP patients had concurrent live abscess or other metastatic infection. All VAP patients received concordant antibiotic treatment according to the results of susceptibility testing. The clinical characteristics of HV-positive and -negative *K. pneumoniae* VAP patients are compared in Table 1. Compared with patients with HV-negative *K. pneumoniae*, those with HV-positive strains had a significantly higher prevalence of cardiac-cerebrovascular disease (55.1 % vs 85.7 %, respectively, *P* = 0.049), a significantly higher prevalence of bacteremic *K. pneumoniae* (3.4 % vs 35.7 %, respectively, *P* = 0.017) and a significantly higher mortality rate (57.1 % vs 13.8 %, respectively, *P* = 0.009). The laboratory data of the *K. pneumoniae* VAP patients is presented in Table 2. Compared with patients with HV-negative *K. pneumoniae*, those with HV-positive strains had a significantly higher C-reactive protein (CRP) levels (100.16 ± 77.62 vs 156 ± 53.89, respectively, *P* = 0.03), lower albumin levels (34.007 ± 6.49 vs 29.68 ± 5.31, respectively, *P* = 0.038).

**Table 1:** Relationship between hypermucoviscosity phenotype of *K. pneumoniae* and clinical characteristics

	Hypermucoviscosity		
Characteristic	Positive	Negative	*P* value
	*n* = 14	*n* = 29	
	No. (%)	No. (%)	
Age mean ± SD	64 ± 14	53 ± 15	0.613
Male sex	11 (78.6)	21 (72.4)	0.665
Underlying disease			
Diabetes mellitus	3 (21.4)	2 (6.8)	0.376
Malignancy	1 (7.1)	1 (3.4)	1.000
Neurologic disorders	6 (42.9)	13 (44.8)	0.903
Trauma	1 (7.1)	5 (17.2)	0.670
Chronic lung disease	1 (7.1)	1 (3.4)	1.000
Cardiac-cerebrovascular disease	12 (85.7)	16 (55.1)	0.049^*^
ICU stay, days	14 ± 6	13 ± 5	0.452
Mechanical ventilation, days	8 ± 4	6 ± 3	0.141
Bacteremia	5(35.7)	1 (3.4)	0.017^*^
Mortality	8 (57.1)	4 (13.8)	0.009^*^

Values given as means ± SD or No. (%) of patients

^*^ *P* < 0.05 was considered to be statistically significant

**Table 2:** Relationship between hypermucoviscosity phenotype of *K. pneumoniae* and laboratory data

	Hypermucoviscosity		
Characteristic	Positive	Negative	*P* value
	*n* = 14	*n* = 29	
	No. (%)	No. (%)	
Chest radiography			
Unilateral involvement	3 (21.7)	6 (20.6)	1.000
Bilateral involvement	11 (78.6)	23 (79.3)	1.000
Initial laboratory value			
Leukocyte count, ×10^9^/l	14.56 ± 7.21	15.65 ± 6.21	0.613
Platelet, ×10^9^/l	139.71 ± 82.27	192.10 ± 130.13	0.177
Albumin, g/l	29.68 ± 5.31	34.007 ± 6.49	0.038^*^
C-reactive protein, mg/l	156.79 ± 53.89	100.16 ± 77.62	0.03^*^
Glucose, mmol/l	9.85 ± 3.92	8.12 ± 2.94	0.115

Values given as No. (%) of patients

^*^ *P* < 0.05 was considered to be statistically significant

### Microbiological characteristics of *K. pneumoniae*

Among 70 *K. pneumoniae* isolates analyzed, 14 (20 %) of them were found to be HV-positive strains and 56 (80 %) of them were HV-negative strains. We further observed that the frequency of VAP was significantly higher in patients with HV-positive strains (100 %; 14/14) than in patients with HV-negative strains (51.8 %; 29/56). The results showed that HV-phenotype was highly correlated with the presence of the *rmpA* and aerobactin genes. Of 14 HV-positive isolates, 85.7 % (12/14) were *rmpA* and aerobactin positive, and 14.3 % (2/14) were *rmpA* and aerobactin negative. None of HV-negative isolates was *rmpA* or aerobactin positive. Serotypes K1 and K2 accounted for 14.3 % (2/14) and 28.6 % (4/14), respectively, of the HV-positive isolates. Serotypes K1 and K2 were not found among HV-negative isolates. All 12 *rmpA*-positive isolates carried the aerobactin gene. All K1/K2 isolates (*n* = 6) were positive for HV-phenotype and the *rmpA* and aerobactin genes (Table 3).

**Table 3:** Microbiological characteristics of *K. pneumoniae* from mechanically ventilated patients

Variable	No. of isolates *n* = 70	Hypermucoviscosity		*P* value
		Positive	Negative	
		*n* = 14	*n* = 56	
		No. (%)	No. (%)	
Capsular serotype				
K1	2	2 (14.3)	0 (0)	0.038^*^
K2	4	4 (28.6)	0 (0)	0.001^*^
Virulence factors				
*rmpA*	12	12 (85.7)	0 (0)	<0.001^*^
aerobactin	12	12 (85.7)	0 (0)	<0.001^*^
VAP due to *K.pneumoniae*	43	14 (100)	29 (51.7)	0.001^*^

Values given as No. (%) of patients

^*^ *P* < 0.05 was considered to be statistically significant

### Antimicrobial susceptibility test

The prevalence of HV-negative isolates exhibiting resistance to the tested antimicrobials was higher than that of the HV-positive isolates (Table 4). The detection rates of ESBL-producing *K. pneumoniae* isolates were 46.5 % (20/43). The percentage of ESBL-producing HV-negative isolates was significantly higher than that of ESBL-producing HV-positive isolates (62.1 % compared to 14.3 %, *P* < 0.05). Among all of the isolates, one of them (1/43, 2.3 %) was resistant to imipenem and meropenem.

**Table 4:** Difference of the antimicrobial susceptibility between HV-positive and- negative of *K. pneumoniae*

	No. (%) of patients susceptible		
Antimicrobial agent	HV-Positive isolates	HV-Negative isolates	*P* value
	*n* = 14	*n* = 29	
piperacillin	8 (57.1)	9 (31.0)	0.101
Cefazolin	5 (35.7)	10 (34.4)	1.000
Cefoxitin	10 (71.4)	14 (48.3)	0.152
Cefuroxime	11 (78.6)	11(37.9)	0.012^*^
Ceftriaxone	12 (85.7)	12 (41.4)	0.006^*^
Ceftazidime	12 (85.7)	12 (41.4)	0.006^*^
Cefotaxime	12 (85.7)	13 (44.8)	0.011^*^
Cefepime	13 (92.9)	23 (79.3)	0.492
Imipenem	14 (100)	28 (96.6)	1.000
Meropenem	14 (100)	28 (96.6)	1.000
Ciprofloxacin	12 (85.7)	15 (51.7)	0.031^*^
Levofloxacin	12 (85.7)	16 (55.2)	0.104
Gentamicin	14 (100)	17(58.6)	0.013^*^
Amikacin	14 (100)	18 (62.1)	0.022^*^
Trimethoprim-sulfamethoxazole	12 (85.7)	17 (58.6)	0.013^*^
ESBLs	2 (14.3)	18 (62.1)	0.003^*^

Values given as No. (%) of patients

ESBL, extended-spectrum β-lactamase

^*^ *P* < 0.05 was considered to be statistically significant

### MLST profiles of isolates from patients with VAP

**Fig. 1:**
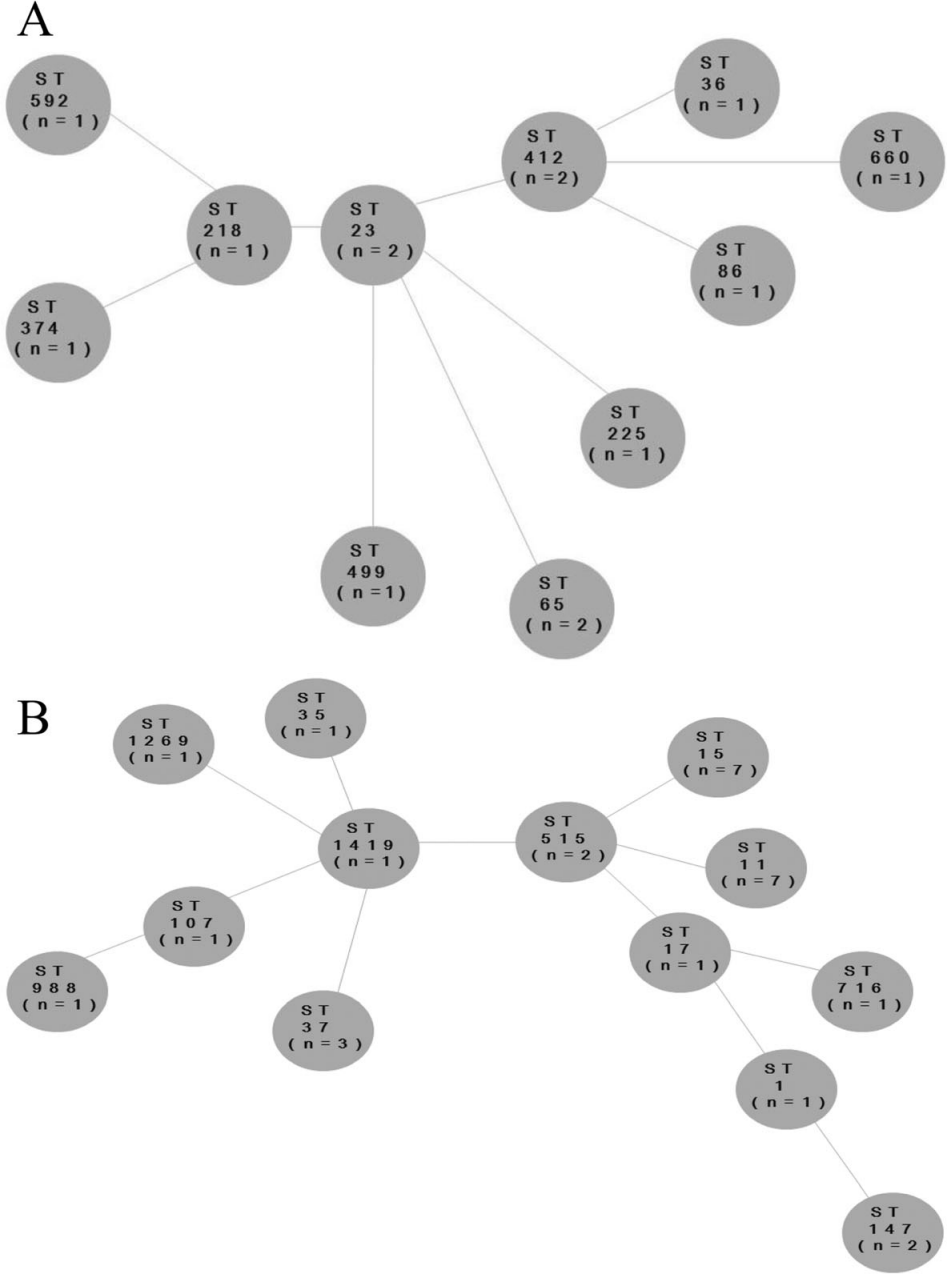
Minimum-spanning tree of 14 isolates of hypermucoviscosity-positive strains (**a**) and 29 hypermucoviscosity-negative strains (**b**) generated with multilocus sequence typing (MLST) allelic data (analysis at MLST website)

Fourteen HV-positive strains and 29 HV-negative strains were isolated from VAP patients. Eleven MLSTs—including sequence types (STs) 23, 36, 65, 86, 218, 225, 374, 412, 499, 592, and 660—were identified among the HV-positive strains. Two major MLST groups, ST218-like and ST23-like, were obtained based on minimum-spanning tree analysis (Fig. 1a). Two serotype K1 isolates belonged to ST23. In addition, three MLSTs—STs 374, 86, and 65—were identified among serotype K2 isolates. All of the remaining HV-positive strains except STs 225 and 499 were positive for *rmpA* and aerobactin. On the contrary, 13 MLSTs—including STs 1, 11, 15, 17, 35, 37, 107, 147, 515, 716, 988, 1269, and 1419—were identified among HV-negative isolates. Minimum-spanning tree analysis showed that all of these isolates belonged to two major MLST groups, ST1419-like and ST515-like (Fig. 1b). ST11 (*n* = 7) and ST15 (*n* = 7) were the prevalent STs in HV-negative isolates.

## Discussion

In the retrospective study, we analyzed 70 isolates of *K. pneumoniae* from mechanically ventilated patients between January 2012 and August 2014. We observed that HV-positive strains accounted for 20 % (14/70) *K. pneumoniae* isolates. The genotypes of K1, K2, *rmpA* and aerobactin were only positive for HV-positive strains. The medical records showed that 43 strains accounted for 61.4 % (43/70) induced VAP in mechanically ventilated patients. There was difference in ST distribution between HV-positive and HV-negative stains in VAP patients.

We further observed that the occurrence rate of VAP was significantly higher for HV-positive strains infection than HV-negative strains infection (100 % vs 51.7 %, *P* < 0.05). Furthermore, the results showed that bacteremic VAP occurred more frequently in HV-positive strains than in HV-negative strains (35.7 % vs 3.4 %, respectively, *P* = 0.017). The mortality rate was higher in the HV-positive group (57.1 %) than in the HV-negative group (13.8 %). Laboratory data also showed that HV-positive strains VAP patients had increased frequency of higher C-reactive protein (CRP) and lower albumin levels. CRP is an acute-phase protein that has been evaluated in the critical care setting [24]. Moreover, CRP values correlated well with the severity of the infection. Hillas et al. [25] observed that a rise in CRP between days 1 and 7 increases the risk of septic shock. Albumin level reportedly decreases as part of negative acute-phase protein. Previous studies have indicated that the degree of hypoalbuminemia in critically ill patients correlates with the intensity of the inflammatory response caused by infection [26, 27]. These findings indicated that compared with HV-negative strains, HV-positive strains are more virulent in the development of VAP.

Capsular serotypes, especially serotypes K1 and K2, are important virulence factors for *K. pneumoniae* [28, 29]. Yu et al. reported that the capsular serotypes K1 and K2 have particular virulence and were more common in patients with community-acquired pneumonia (23/49 isolates, 47 %) than in those with hospital-acquired pneumonia (2/18 isolates, 11 %) [30]. Our results were similar to the study. We also found that capsular serotypes K1 and K2 comprised only 4.7 % (2/43) and 9.3 % (4/43), respectively, of all *K. pneumoniae* isolated from VAP patients. In addition, the HV-positive strains accounted for 32.6 % (14/43) *K. pneumoniae* in VAP patients. These results suggest that hypermucoviscosity rather than serotype K1 or K2 has become a common pathogen of VAP in mechanically ventilated patients.

Previous study has shown that K1 serotype and *rmpA* are associated with HV-phenotype in *K. pneumoniae* [17]. In this study, we found that HV-phenotype frequently coexists with the *rmpA* gene. In addition, the prevalence of the K1 serotype in HV-positive strains was only 14.3 % (2/14). Chen et al. further confirmed that the *rmpA* gene is a regulator of HV-phenotype [31]. Loss of this gene leads to the loss of HV-phenotype. These data showed that the *rmpA* gene rather than the K1 serotype correlated with HV-phenotype in *K. pneumoniae*. Meanwhile, the results also showed that two HV-positive strains which did not possessed *rmpA* gene. This indicated that there may be other regulatory mechanisms for expression of HV-phenotype.

In addition, we found that *rmpA* gene coexists with the aerobactin gene in HV-positive *K. pneumoniae*. This result was in line with the observations of Yu et al. [30]. Further investigation showed that *rmpA* is located on a 180-kb virulence plasmid, which also contains many virulence-associated genes, including aerobactin for iron acquisition [15, 32]. However, we did not determine which of the two virulence factors (*rmpA* or aerobactin) of HV-positive strains is more critical in VAP.

Among these VAP patients, the HV-positive isolates were significantly more susceptible to the antimicrobial agents that were tested, when compared with the HV-negative isolates. However, we found two ESBL-producing HV-positive isolates. Previous study also showed that most HV-positive strains were very susceptible to antimicrobials [33]. Nonetheless, some cases of infection due to multidrug resistant HV-positive *K. pneumoniae* have already been described [34]. Therefore, management of VAP due to HV-positive isolates will become extremely challenging.

In the present study, 24 STs were observed in the 43 *K. pneumoniae* isolates from VAP patients. Eleven MLSTs were identified in 14 HV-positive strains, suggesting a polyclonal origin. Thirteen STs were observed in 29 HV-negative strains, among which ST11 (*n* = 7) and ST15 (*n* = 7) were the most prevalent. We noticed that a specific ST distribution occurred between HV-positive and HV-negative strains, suggesting the difference genetic background existed among the 2 phenotype isolates.

This study had several limitations. First, it was a retrospective study from a single hospital and the small sample size may have had selection bias. Second, the diagnosis of VAP in mechanically ventilated patients is difficult, and still there is no “gold-standard” diagnostic method. Third, the pathogenic mechanism of HV-positive *K. pneumoniae* in VAP is unclear and requires further investigation in future studies.

## Conclusion

We have shown that HV-phenotype, rather than serotype K1 or K2, was the major virulence determinant for mechanically ventilated patients. Patients infected with HV-positive strains were more likely to develop VAP and bacteremic VAP. Furthermore, HV-positive *K. pneumoniae* VAP had a higher mortality than HV-negative strains VAP. We hope our results will draw attention from physicians, which may lead to prompt recognition and successful management of HV-positive *K. pneumoniae* VAP.

## Abbreviations

ESBL: Extended-spectrum β-lactamase
HV: Hypermucoviscosity
ICU: Intensive care units
MLST: Multilocus sequence typing
VAP: Ventilator-associated pneumonia.
